# Fos expression in the orbital prefrontal cortex after exposure to the fixed-interval peak procedure

**DOI:** 10.1016/j.bbr.2012.01.035

**Published:** 2012-04-15

**Authors:** L. Valencia-Torres, C.M. Olarte-Sánchez, S. Body, K.C.F. Fone, C.M. Bradshaw, E. Szabadi

**Affiliations:** a Psychopharmacology Section, Division of Psychiatry, University of Nottingham, Room B109, Medical School, Queen's Medical Centre, Nottingham NG7 2UH, UK; b School of Biomedical Sciences, University of Nottingham, Room E20, Medical School, Queen's Medical Centre, Nottingham NG7 2UH, UK

**Keywords:** Orbital prefrontal cortex, Corpus striatum, Fos expression, Interval timing behaviour, Temporal differentiation, Fixed-interval peak procedure, Variable-interval schedule, Rat

## Abstract

It has been proposed that cortico-striato-thalamo-cortical circuits that incorporate the prefrontal cortex and dorsal striatum regulate interval timing behaviour. The present experiment examined whether performance on the fixed-interval peak procedure (FIPP), an immediate timing schedule, would induce neuronal activity in cortical and striatal areas, as revealed by enhanced expression of the Fos protein, a marker for neuronal activation. Regional Fos expression was compared between rats trained on the FIPP and rats trained on a variable-interval (VI) schedule matched to the FIPP for overall response rate and reinforcer delivery. Response rate in the peak trials of the FIPP conformed to a temporally differentiated pattern, which was well described by a modified Gaussian function; in agreement with previous findings, the peak time occurred close to the time at which the reinforcer was delivered in the fixed-interval trials, and the Weber fraction was within the range of values reported previously. The density of Fos-positive neurones (counts mm^−2^) in the orbital prefrontal cortex (OPFC) was greater in rats exposed to the FIPP than in rats exposed to the VI schedule, suggesting a greater activation of this area during the performance of the former task. This is consistent with the results of previous studies that have implicated the OPFC in interval timing behaviour. However, there was no significant difference between the levels of Fos expression in the dorsal or ventral striatum of the rats trained under the two schedules.

## Highlights

► The effect of timing behaviour on cortical and striatal Fos expression was examined. ► Rats trained on the peak procedure (FIPP) and a control schedule were compared. ► Fos expression in the orbital PFC was enhanced following exposure to the FIPP. ► Fos expression in the striatum did not differ between rats trained on the two tasks. ► The results suggest that the orbital PFC is involved in interval timing behaviour.

## 1 Introduction

Timing behaviour plays an important role in the daily living of individuals from a wide variety of species. Animals must be able to discriminate between the durations of relevant events in their environments (temporal discrimination) and to regulate their own behaviour in time (temporal differentiation). Temporal discrimination is revealed by retrospective timing schedules such as the interval bisection task, and temporal differentiation by immediate timing tasks such as the fixed-interval peak procedure (FIPP) [1]. The FIPP is composed of two types of trial that are presented in random sequence. In standard fixed-interval trials, a reinforcer is delivered following the first response that the animal emits after a fixed interval has elapsed since the start on the trial. Peak trials are usually three or four times longer than the fixed-interval trials, and no reinforcers are delivered in these trials. Response rate during peak trials has been found to rise to a maximum around the time of reinforcement in the fixed-interval trials (the peak time), and then to decline; a secondary rise in response rate is often seen towards the end of the trial [2].

A growing body of evidence supports the involvement of cortico-striato-thalamo-cortical circuits in interval timing [3,4]. For instance, it has been reported that there is an increase in the firing rate of striatal and cortical neurones recorded in rats during performance of a multiple-duration fixed-interval procedure [5], and lesions of the dorsal striatum in rats have been found to disrupt timing on the FIPP [6]. Electrophysiological and functional imaging studies in humans have also demonstrated that the dorsal striatum is activated during performance of time discrimination tasks [7–10].

Valencia-Torres et al. [11] recently examined Fos expression, a marker of neuronal activation, in the prefrontal cortex and corpus striatum during performance of temporal discrimination tasks. It was found that rats trained under temporal discrimination tasks showed increased Fos expression in the prefrontal cortex and ventral striatum (nucleus accumbens), compared to rats trained under non-temporal (light-intensity) discriminaton tasks, suggesting that these areas are activated during interval timing performance. Interestingly, Fos expression was not enhanced in the dorsal striatum during performance of the temporal discrimination tasks, an unexpected result in view of the evidence for a pivotal role of the dorsal striatum in interval timing [3–6]. Valencia-Torres et al. [11] noted that while their experiments employed retrospective timing tasks, most of the studies that had demonstrated a link between interval timing and the dorsal striatum employed immediate timing tasks such as the FIPP [3–6]. Valencia-Torres et al. suggested that the apparent discrepancy between their results and previous findings that implicated the dorsal striatum in interval timing might have been due to the particular timing tasks used in different studies. In order to address this possibility, the present study examined the pattern of Fos expression in the prefrontal cortex and corpus striatum following exposure to the FIPP.

Fos is the protein product of the immediate-early gene *c-Fos* which is found in neuronal nuclei. In most neurones, Fos levels are low under basal conditions, but neuronal firing results in an increase in Fos production [12]. Changes in Fos expression may therefore act as a biomarker for relatively short-term changes in neuronal activity induced by physiological or behavioural manipulations [13,14].

Fos expression may be induced by various aspects of operant behavioural tasks that are not directly related to the process of primary interest, for example, food deprivation, food consumption [15,16] and locomotor activity [17–19]. For this reason, it is important that any experiment in which Fos expression is used as a means of identifying the neural structures involved in particular behavioural processes should employ a control procedure that is matched as closely as possible to the index task on these ‘irrelevant’ variables. In the present experiment, the pattern of Fos expression in the prefrontal cortex and corpus striatum was compared between a group of rats trained under the FIPP and a control group trained under a variable-interval (VI) schedule [20] that entailed the same food deprivation conditions, the same response requirements and the same overall reinforcement rate as the FIPP. In a VI schedule, reinforcement follows the first response that the subject emits after a variable time has elapsed since the previous reinforcer. Unlike the FIPP, reinforcer availability in a VI schedule does not follow a regular temporal pattern, and it is therefore assumed that temporal differentiation is not involved in performance on this schedule [20,21].

## 2 Methods

The experiment was carried out in accordance with UK Home Office regulations governing experiments on living animals, and was approved by the University of Nottingham Ethical Review Committee.

### 2.1 Subjects

Twenty-four experimentally naive female Wistar rats (Charles River, UK) aged approximately 4 months and weighing 250–300 g at the start of the experiment were used. They were housed individually under a constant cycle of 12 h light and 12 h darkness (light on 06:00–18:00 h), and were maintained at 80% of their initial free-feeding body weights throughout the experiment by providing a limited amount of standard rodent diet after each experimental session. Tap water was freely available in the home cages.

### 2.2 Apparatus

The rats were trained in operant conditioning chambers of internal dimensions 20 cm × 23 cm × 22.5 cm (Campden Instruments Ltd., UK). One wall of the chamber contained a recess into which a motor-operated dispenser could deliver food pellets (TestDiet, MLab Rodent Tablet 45 mg; Sandown Scientific, UK). Apertures were situated 5 cm above and 2.5 cm on either side of the recess; motor-operated retractable levers could be inserted into the chamber through these apertures. The levers could be depressed by a force of approximately 0.2 N. The chamber was enclosed in a sound-attenuating chest; masking noise (approximately 80 dB[A]) was provided by a rotary fan. An Acorn 600 microcomputer and interface unit (Paul Fray Ltd., UK), programmed in ARACHNID BASIC and located in an adjoining room, controlled the schedules and recorded the behavioural data. Only one lever was used in this study; this was the left-hand lever for half the rats and the right-hand lever for the other half.

### 2.3 Behavioural training

The rats were allocated into two groups that were trained under the FIPP (*n* = 12) or VI (*n* = 12) schedule. At the start of the experiment, the food-deprivation regimen was started and the rats were gradually reduced to 80% of their free-feeding body weights. They were then trained to press the lever by providing reinforcers intermittently, in the absence of the lever, for three sessions (50 reinforcers per session), followed by three sessions of exposure to a discrete-trials continuous reinforcement schedule, in which one lever was presented intermittently, and a single response resulted in retraction of lever and delivery of a food pellet. Thereafter, the rats underwent 45-min training sessions on the FIPP or VI schedule, as described below, 7 days a week at the same time each day during the light phase of the daily cycle (between 08.00 and 13.00 h), for a total of 60 sessions.

*Fixed-interval 30-s peak procedure* (*FIPP 30-s*) (*n* = 12). Each session consisted of 48 trials separated by 10-s inter-trial intervals. Trials started with insertion of the lever into the chamber, and terminated with lever withdrawal. In fixed-interval trials (32 per session), reinforcement was delivered following the first response emitted after 30 s had elapsed since the onset of the trial. The lever was withdrawn and the trial terminated when a reinforcer was delivered; if no response occurred within 5 s of the reinforcer becoming available, the trial was terminated without reinforcer delivery. In peak trials (16 per session), reinforcement was omitted, and the lever remained in the chamber for 120 s. The fixed-interval and peak trials occurred in a pseudo-random sequence with the constraint that no more than three trials of either type occurred in succession. Timing behaviour was assessed from performance in the peak trials (see below).

*Variable-interval 75-s schedule* (*VI 75-s*) (*n* = 12). Each session consisted of 48 trials separated by 10-s inter-trial intervals (32 30-s trials and 16 120-s trials). As in the FIPP, trials started with insertion of the lever into the chamber, and terminated with lever withdrawal. A constant-probability VI 75-s schedule based on the sequence of intervals described by Catania and Reynolds [21] was operative throughout all the trials, pausing only during reinforcer presentation (5 s) and the inter-trial intervals (10 s). The mean inter-reinforcer interval specified by the schedule (75 s) was chosen empirically in order to equate, as closely as possible, the overall rate of reinforcer delivery in the FIPP and the VI schedule. The maximum reinforcement rate specified by the schedule was approximately 36 reinforcers per session; the obtained reinforcement rate was approximately 30 reinforcers per session (see below).

### 2.4 Immunohistochemistry

Ninety minutes after the final session animals were perfused transcardially with phosphate-buffered physiological saline (PBS) (0.1 M) followed by 4% paraformaldehyde in PBS (formol PBS) under deep anaesthesia with sodium pentobarbitone. Brains were removed and fixed in formol PBS. After 4 h, they were transferred to 30% sucrose solution for 48 h. Forty-micrometer-thick coronal sections were cut on a freezing microtome. Free-floating sections were washed in PBS and then treated with 0.3% H_2_O_2_ in PBS for 30 min. Subsequently, the sections were treated with a blocking solution containing 3% normal goat serum (NGS) and 0.3% Triton-X in PBS, and incubated for 2 days at 4 °C with the primary antibody [polyclonal anti-Fos protein raised in rabbit (Santa Cruz Biotechnology, Santa Cruz, CA, USA), 1:5000 dilution in PBS containing 3% NGS and 0.3% Triton-X]. This was followed by incubation in the secondary antibody, biotinylated goat anti-rabbit IgG (Vector Laboratories, Burlingame, CA, USA; 1:600), for 2 h, and by incubation with peroxidase-conjugated avidin–biotin complex (Vector Laboratories) for 1 h. The reaction was developed with 3,3′-diaminobenzidine. The sections were mounted on chrome-gelatin-coated microscope slides and dehydrated in graded alcohols (70%, 80%, 90%, and 100% ethanol), cleared in xylene and coverslipped with DPX.

Fos-positive nuclei were identified by the dark reaction product confined to the nucleus and quantified from digital images of sections at a magnification of 50× (ImageJ software; Wayne Rasband, National Institutes of Health, USA). The brain structures were outlined according to Paxinos and Watson's [22] stereotaxic atlas. The areas analysed were the following: infralimbic (ILPFC), prelimbic (PLPFC) and orbital (OPFC) prefrontal cortex, the core of the nucleus accumbens (AcbC), the medial and lateral portions of the shell of the nucleus accumbens (AcbS) and the dorsomedial (DMCP) and dorsolateral (DLCP) caudate-putamen (see Fig. 2, right-hand panels for definition of the regions).

### 2.5 Data analysis

*Behavioural data.* The data from each rat, averaged across the last ten training sessions, were used in the analysis. Response rates in successive 2-s epochs of the 120-s trials (in the case of the FIPP, these were the peak trials, see above) were plotted against time measured from the start of the trial; a two-factor analysis of variance was carried out on the data (group × time-bin, with repeated measures on the latter factor). The total numbers of responses emitted and reinforcers obtained per session were compared between the two groups using Student's *t*-test. In the case of the FIPP 30-s group, the following modified Gaussian function (‘Gaussian plus ramp’ function [24]) was fitted to each rat's response rate data in the peak trials:

$$R=a\times e^{\left[-0.5\times {\left(\frac{t-t_{\text{peak}}}{b}\right)}^{2}\right]}+[c+d\times (t-t_{\text{peak}})]\text{,}$$

where (*a* + *c*) is the estimated peak response rate, *t*_peak_ is the peak time (location of the peak of the Gaussian component of the function), *b* represents the spread of the function (standard deviation of the Gaussian component); the right-hand term is a linear ramp of slope *d* and an ordinate value *c* at time *t* = *t*_peak_. This function has been found to provide an acceptable description of performance in the FIPP [23–25]. The following measures were derived for each rat: the peak time (*t*_peak_), the peak response rate (*a* + *c*), and the Weber fraction (coefficient of variation of the Gaussian component of the function: *b*/*t*_peak_). Goodness of fit of the fitted functions was expressed as *r*^2^.

*Immunohistochemical data*. Fos-positive nuclei were quantified as described above. The number of Fos-positive nuclei in each area was compared between groups by multivariate analysis of variance (MANOVA); partial *η*^2^ was used as the measure of effect size.

A significance criterion of *p* < 0.05 was used in all statistical analyses.

## 3 Results

One subject in the VI 75-s group was removed from the analysis because it failed to develop stable response rates within trials. The analyses were therefore based on data from 12 rats trained under the FIPP 30-s and 11 rats trained under the VI 75-s schedule.

### 3.1 Behavioural data

**Fig. 1:**
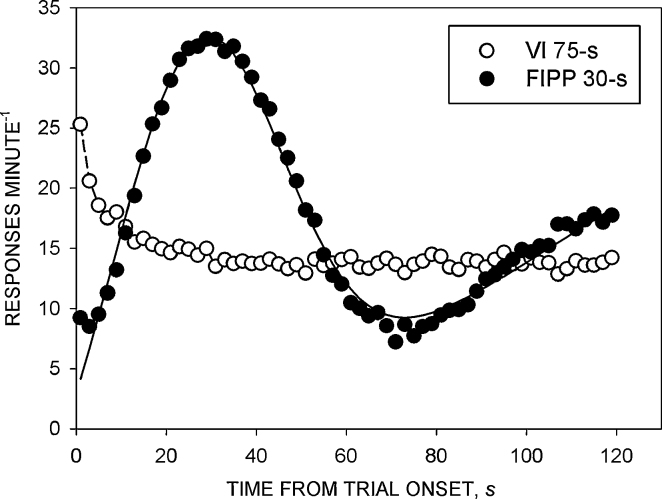
Comparison of performance on the fixed-interval peak procedure (FIPP) 30-s and the variable-interval (VI) 75-s schedule. Ordinate: response rate (responses minute^−1^); abscissa: time from trial onset (s). Points are group mean data from successive 2-s time-bins in the last ten sessions of the experiment: open circles, rats trained under VI 75-s; filled circles, rats trained under FIPP 30-s. The continuous curve is the best-fit modified Gaussian function for the rats trained under FIPP 30-s.

*Timing performance on the FIPP 30-s schedule.* The group mean data obtained in the peak trials are shown in Fig. 1 (filled circles). The peak of the response rate function occurred close to the time at which reinforcement became available in the FI trials. Table 1 shows the group mean (±SEM) values of the timing parameters derived from the fits of the modified Gaussian function to the data from the individual rats. The peak time (*t*_peak_) (26.1 s) was close to the scheduled reinforcement time (30 s) and the Weber fraction was approximately 0.76. The function accounted for approximately 90% of the data variance from the individual rats and 98% of the variance of the group mean data.

*Comparison of performance on the FIPP 30-s and VI 75-s schedules.* Fig. 1 (open circles) shows the response rates of the rats trained under the VI 75-s schedule. Response rate tended to be highest at the start of the trial, and then to decline over approximately 8–10 s to a level that was maintained until the end of the trial. Analysis of variance of the response rate data showed no significant main effect of group [*F* < 1], but a significant main effect of time-bin [*F*(59, 1239) = 10.4, *p* < 0.001] and a significant group × time-bin interaction [*F*(159, 1239) = 12.5, *p* < 0.001]. Table 2 shows the mean number of responses and reinforcers obtained during the last ten sessions. There was no significant difference between either the total number of responses [*t*(21) = 0.9, NS] or the total number of reinforcers [*t*(21) = 0.9, NS] of the two groups, indicating that the groups were appropriately matched on these two measures.

### 3.2 Immunohistochemical data

**Fig. 2:**
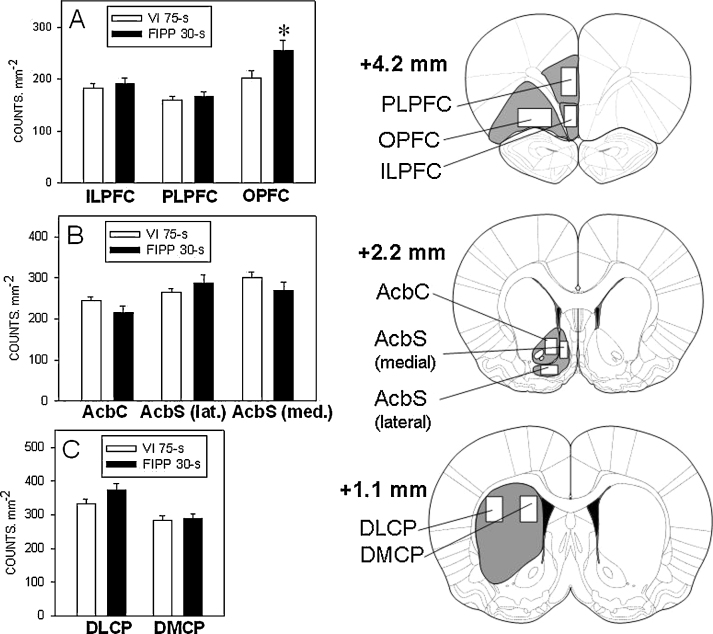
*Left-hand panels.* Density of Fos-positive units (counts mm^−2^) counted in the cortical and striatal regions: (A) infralimbic (ILPFC), prelimbic (PLPFC) and orbital (OPFC) prefrontal cortex; (B) nucleus accumbens core (AcbC) and medial and lateral portions of the nucleus accumbens shell (AcbS); (C) dorsomedial (DMCP) and dorsolateral (DLCP) caudate-putamen. Columns show the group mean data (+SEM) in each area for the rats trained under the variable-interval (VI) 75-s schedule (empty columns) and the fixed-interval peak procedure (FIPP) 30-s (filled columns). Significant difference between the groups, * *p* < 0.05. *Right-hand panels*. Diagramatic representation of the areas selected for counting Fos-positive units (three coronal sections at AP locations, measured from bregma, as indicated; modified from Paxinos and Watson [22]).

**Fig. 3:**
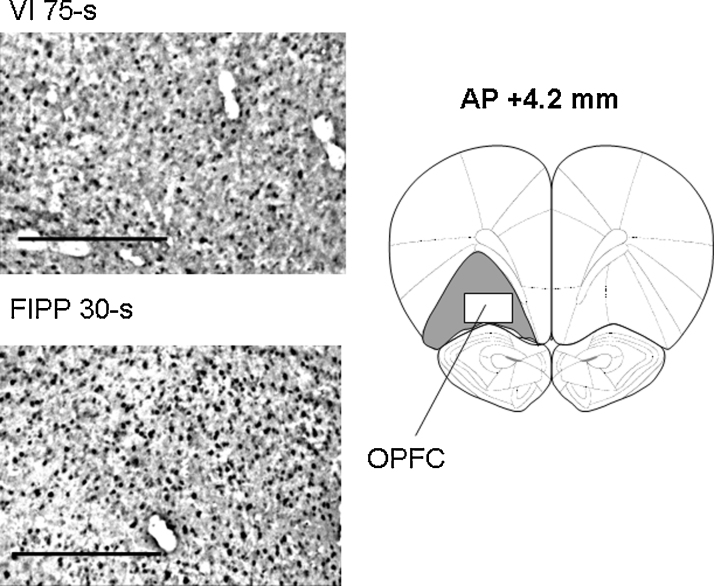
*Left-hand panels.* Examples of Fos expression in the orbital prefrontal cortex (OPFC) in a representative rat trained under the variable-interval (VI) 75-s schedule (upper panel) and the fixed-interval peak procedure (FIPP) 30-s (lower panel). The bar represents 1 mm. *Right-hand panel.* Diagramatic representation of the area selected for counting Fos-positive units (AP location, measured from bregma, as indicated; modified from Paxinos and Watson [22]).

Fig. 2 shows the group mean (±SEM) numbers of Fos-positive neurones in cortical and striatal areas of the rats trained under the FIPP 30-s and VI 75-s schedules. MANOVA revealed a significant difference between the two groups in the OPFC [*F*(1, 20) = 4.8, *p* < 0.05, partial *η*^2^ = 0.19], higher levels of Fos expression being seen in the rats trained under the FIPP 30-s than in those trained under the VI 75-s schedule. There was no significant between-group difference in Fos expression in the ILPFC or PLPFC or in any of the ventral or dorsal striatal areas examined. (The level of Fos expression in the DLCP was somewhat higher in the rats trained under the FIPP than in those trained under the VI schedule; however the difference fell short of statistical significance [*F*(1, 20) = 2.7, *p* = 0.12, partial *η*^2^ = 0.11].) Representative examples of coronal sections showing Fos expression in the OPFC of a rat from each experimental group are given in Fig. 3.

## 4 Discussion

Performance on the FIPP was similar to that reported in many previous studies [2,24–28]. Response rate increased to a maximum at approximately the scheduled reinforcement time and then decreased in a more or less symmetrical way; a secondary rise in response rate occurred towards the end on the trial [24,25]. Performance was well described by the modified Gaussian function. The peak time and the Weber fraction were within the range of values reported in previous studies [2,25–27].

Performance under the VI schedule was similar to that reported in previous studies [21]. Response rate declined rapidly from an initially high level at the start of the trial, and a relatively constant rate of responding was maintained throughout the remainder of the trial. The initial decline of response rate has been observed previously in experiments in which VI schedules have been presented in discrete trials [28–31], and has been attributed to a reduction of arousal from a high level occasioned by the initial presentation of the operandum at the start of the trial [32].

Analysis of response rates under the two schedules indicated that, as expected, the temporal differentiation of responding seen in the rats trained under the FIPP was absent in the rats trained under the VI schedule. However, the overall numbers of responses and reinforcers per session did not differ significantly between the groups.

The density of Fos-positive neurones (counts mm^−2^) in the OPFC was greater in the rats exposed to the FIPP than in those exposed to the VI schedule, suggesting a greater activation of this area during the performance of the former task. This difference in Fos expression between the two groups is unlikely to have been caused by differences in reinforcer consumption or repetitive execution of the operant response, since the control group trained under the VI schedule underwent the same food deprivation conditions as the group trained under the FIPP, and the numbers of responses emitted and reinforcers obtained did not differ between the two groups. The present result is thus consistent with the notion that neuronal activation in the OPFC was related to the temporal control of behaviour exerted by the FIPP. This is in agreement with the results of previous studies that have implicated the prefrontal cortex in temporal differentiation. For example, it has been reported that lesions of different subregions of the frontal cortex can disrupt timing performance or slow the acquisition of timing behaviour of rats on the FIPP [33,34]. It should be noted that the involvement of the OPFC in interval timing seems not to be restricted to temporal differentiation, since Valencia-Torres et al. [11] observed enhanced Fos expression in the OPFC associated with temporal discrimination performance in retrospective timing schedules (e.g. the interval bisection task).

Peformance on the FIPP was not associated with significantly enhanced Fos expression in the dorsal striatum, compared to the control (VI) schedule. Although the level of Fos expression in the DLCP was somewhat higher in rats trained under the FIPP than in those trained under the VI schedule; however the difference did not attain statistical significance. Thus the present results do not provide any compellinig evidence for a specific involvement of the dorsal striatum in FIPP performance. This negative result was unexpected, because a considerable body of evidence has implicated the dorsal striatum in interval timing behaviour. Lesions of the dorsal striatum have been found to disrupt performance on the FIPP [6], and the firing rate of striatal neurones recorded during the performance of a temporal generalization task has been found to increase progressively towards a peak at the expected time of reinforcement [5]. It is possible that the lack of a significant effect of the timing task on striatal Fos expression in the present experiment may reflect the relatively high rates of operant responding seen in the rats trained under both schedules. There is evidence that locomotor activity is associated with enhanced Fos expression in the striatum [17], and it is possible, therefore, that any increment in Fos expression induced by the timing contingency may have been masked by a larger increment induced by non-temporal motor aspects of schedule performance.

There was no significant difference between the levels of Fos expression in the nucleus accumbens of the two groups. In this respect the present results differ from those of Valencia-Torres et al. [11], who reported that rats performing temporal discrimination tasks showed higher levels of Fos expression in the nucleus accumbens than rats trained under control (light-intensity) discrimination tasks. It is possible that this reflects the engagement of different neural structures by temporal discrimination and temporal differentiation behaviours. However, the possibility cannot be excluded that the greater motor output generated in the free-operant tasks used in the present experiment may have masked an effect of the timing task on Fos expression in the nucleus accumbens (see above).

Another type of operant task in which timing processes may be involved is inter-temporal choice, in which organisms choose between reinforcers that differ with respect to size and delay [35]. The hypothetical process of delay discounting, whereby reinforcing outcomes are deemed to be devalued by the delay interposed between the choice response and the delivery of the primary reinforcer, is widely assumed to imply the operation of a timing process [36]. Indeed, according to one taxonomy of timing schedules, inter-temporal choice tasks are classified as ‘prospective timing schedules’ [1]. It is therefore of interest to consider whether the same neural structures may be involved in inter-temporal choice as in conventional interval timing schedules. Since in most inter-temporal choice protocols the effects of delay are confounded by the effects of reinforcer size [37], inter-temporal choice protocols that distinguish between the effects of delay and magnitude of reinforcement [37] are of particular interest in attempts to identify the neural underpinnings of delay discounting. The OPFC has been linked both to interval timing [11,29,34,37,38] and to inter-temporal choice behaviour [39–43]. Evidence from the effects of lesions of the OPFC [41,42] and Fos expression [43] in the OPFC induced by inter-temporal choice tasks implicate this structure in the processes of both delay discounting and sensitivity to reinforcer size. A considerable body of evidence also favours a significant role of the nucleus accumbens in delay discounting [44], but unlike the OPFC, the nucleus accumbens appears not to be involved in sensitivity to reinforcer size [43,45–47]. Evidence for the involvement of the nucleus accumbens in conventional interval timing schedules is mixed. Lesions of the nucleus accumbens have been found to disrupt performance on the FIPP, but this has been attributed to a specific impairment of sensitivity to the omission of reinforcers in the peak trials rather than to a deficit of interval timing processes per se [48]. Performance on retrospective timing schedules has been found to be associated with enhanced Fos expression in the nucleus accumbens [11]; however, in the present experiment, employing the FIPP, no such effect was observed. Clearly, much further work is needed in order to clarify the neural bases of interval timing and inter-temporal choice. At the present time, all that can be stated with confidence is that the OPFC apparently contributes to the regulation of both types of behaviour. The possible involvement of different regions of the dorsal and ventral striatum in interval timing warrants much further investigation.

In conclusion, the present finding that performance on the FIPP was associated with enhanced Fos expression in the OPFC is consistent with the results of previous studies implicating this structure in the regulation of different forms of voluntary timing behaviour.

## Figures and Tables

**Table 1:** Group mean (±SEM) values of the timing parameters derived from the modified Gaussian function fitted to the data from the individual rats during the last ten sessions of training under the FIPP 30-s.

*t*_peak_ (s)	SD of the Gaussian component	Weber fraction	*r*^2^	Peak response rate (responses min^−1^)
26.1 ± 1.7	18.7 ± 0.9	0.76 ± 0.10	0.90 ± 0.02	38.7 ± 9.7

**Table 2:** Group mean numbers of responses and reinforcers (±SEM) obtained during the last ten sessions of training under the FIPP 30-s and VI 75-s schedules.

Performance measure	Schedule	
	FIPP 30-s	VI 75-s
Total responses per session	1187.5 ± 225.4	952.7 ± 98.9
Total reinforcers per session	29.6 ± 0.1	30.0 ± 0.5
